# TVGG Dental Implant Identification System

**DOI:** 10.3389/fphar.2022.948283

**Published:** 2022-08-08

**Authors:** Jianbin Guo, Pei-Wei Tsai, Xingsi Xue, Dong Wu, Qui Tran Van, Chanaka Nimantha Kaluarachchi, Hong Thi Dang, Nikhitha Chintha

**Affiliations:** ^1^ Fujian Key Laboratory of Oral Diseases and Fujian Provincial Engineering Research Center of Oral Biomaterial and Stomatological Key Lab of Fujian College and University, School and Hospital of Stomatology, Fujian Medical University, Fuzhou, China; ^2^ Department of Computing Technologies, Swinburne University of Technology, Hawthorn, VIC, Australia; ^3^ Fujian Provincial Key Laboratory of Big Data Mining and Applications, Fujian University of Technology, Fuzhou, China; ^4^ Institute of Stomatology and Research Center of Dental and Craniofacial Implants, School and Hospital of Stomatology, Fujian Medical University, Fuzhou, China

**Keywords:** VGG, CNN, dental implant, radiography image, implant identification

## Abstract

Identifying the right accessories for installing the dental implant is a vital element that impacts the sustainability and the reliability of the dental prosthesis when the medical case of a patient is not comprehensive. Dentists need to identify the implant manufacturer from the x-ray image to determine further treatment procedures. Identifying the manufacturer is a high-pressure task under the scaling volume of patients pending in the queue for treatment. To reduce the burden on the doctors, a dental implant identification system is built based on a new proposed thinner VGG model with an on-demand client-server structure. We propose a thinner version of VGG16 called TVGG by reducing the number of neurons in the dense layers to improve the system’s performance and gain advantages from the limited texture and patterns in the dental radiography images. The outcome of the proposed system is compared with the original pre-trained VGG16 to verify the usability of the proposed system.

## 1 Introduction

Oral radiography images are widely used in assisting dentists in making judgements on a patient’s case, evaluating the conditions, and deciding on further treatment or operations that work the best for the patient. One of the essential steps in observing the oral radiography image is that the dentist needs to identify which manufacturer is the one who manufactured the particular implant that has been placed in the patient’s mouth. Identifying the correct implant manufacturer is vital because different implant manufacturers have different operating procedures and corresponding accessories for their products. Using the right supplements and operating procedures ensures the dental prosthesis’s sustainability and reliability. Thus, identifying the dental implant manufacturer from either the intraoral or the extraoral x-ray image is the key to ensuring the quality of the work.

An intraoral x-ray image is obtained by having a film positioned in the buccal cavity. Unlike the intraoral x-ray image, an extraoral x-ray image is obtained by positioning the patient between the x-ray source and the radiographic film. The intraoral technique produces images focusing on a local region of the mouth, but the extraoral approach provides panoramic x-ray images of the mouth. Either way, the dentist needs to identify the manufacturer by observing the implant’s characteristics (Saghiri et al., 2020), shapes (Guarnieri et al., 2019), and patterns (Makary et al., 2019) from the x-ray image and judge from which manufacturer the implant should be. This can be a high-pressure task when a large volume of patients are pending in the queue for proper treatments. The chance of humans making mistakes under a high-pressure scenario is much greater than average. Having a support system assisting the dentists in identifying the implant’s manufacturer is ideal for lifting the burden on the dentists as a solution for this. Hence, many machine learning-based support systems for identifying the dental implant’s manufacturer and related usability studies have been proposed in recent years.

The remaining of this article is organised as follows: the related works are discussed in Section 2, the proposed system model is revealed in Section 3, the experiments and results are summarized and discussed in Section 4 and is followed by the conclusion in Section 5.

## 2 Related Works

Training a deep neural network from scratch is heavily resource consuming. To avoid getting the model with the hard way, using transfer learning to adjust the model based on a pre-trained model is a popular solution. For example, Kim et al. test a set of transfer learning-based systems for identifying the dental implant in 2020 (Kim et al., 2020). They conclude that the tested convolutional neural network (CNN) models can properly classify four dental implants from manufactors of “Brånemark Mk TiUnite,” “Dentium Implantium,” “Straumann Bone Level,” and “Straumann Tissue Level” with high accuracy. Some data augmentation techniques are applied to their collected data set for preventing overfitting. Their experimental results are produced by SquuezeNet (Iandola et al., 2016), GoogLeNet (Szegedy et al., 2015), ResNet (He et al., 2015), and MobileNet-v2 (Sandler et al., 2018). All models used in their work are pre-trained by ImageNet. However, image sources collected in ImageNet are natural images. The pattern and details contained in those images are very different from and much more complex than those in medical radiography images. Using a thinner network structure may already be sufficient for the dental implant identification task. The terminology “thinner” refers to a network layer with less neurons and thus the width of a layer is narrower. A thinner network structure can save much more resources and computational cost in training and model deployment.

Sukegawa et al. compare the dental implant classification results obtained by the basic CNN with three convolution layers, the VGG16 and the VGG19 models (Sukegawa et al., 2020). According to their findings, the classification accuracy before fine-tuning the VGG models is already higher than the basic CNN model. The accuracy is lifted to above 90% after the fine-tuning for both VGG16 and VGG19. This result shows the advantage of using VGG model over the conventional CNN. In 2015, Simonyan and Zisserman conclude that having up to 19 weight layers of the CNN structure is sufficient for the classification accuracy on the ImageNet challenge dataset (Simonyan and Zisserman, 2015). Nevertheless, fine-tuning a deep learning model is highly resource consuming. Lighten the model to accommodate the radiography images may be a more efficient solution.

In 2020, Almubarak et al. propose a two-stage mask R-CNN model for decomposing the object identification task into the object cropping task and the object classification task in a sequence (Almubarak et al., 2020). Their approach utilizes the bounding box and the semantic segmentation output from the mask R-CNN (He et al., 2020) to locate the target for cropping in the first stage. The cropped target is sent to the second stage as the input for classification. The drawback of this method is that it requires the annotation mask to indicate the ground truth for training. The annotation is highly labor demanded and thus is less preferred in many applications. Besides, the quality of the annotation is vital to the model accuracy. Keeping the annotation in the same quality level is also challenging.

Vuola et al. utilize the ensemble learning technique to aggregate the output from a mask R-CNN and a U-Net (Ronneberger et al., 2015) in the nuclei segmentation application (Vvola et al., 2019). Their finding indicate that the mask R-CNN and the U-Net models make mistakes in different parts of the input image. Thus, using ensemble learning to integrate their output provides a better result in the nuclei segmentation task. Although their experiment is carried out with the fluorescence and histology images, similar experience is potential to be taken in use in the dental implant application as well.

In 2022, Liu et al. use R-CNN to detect marginal bone loss around dental implants (Liu et al., 2022). They find that their model output performs similarly to the resident dentist but is less accurate than the experienced dentist. The reason could be that the model is yet fine-tuned. With proper fine-tuning and optimization, the model is expected to be improved. Moreover, R-CNN model has been improved from the original version to the fast R-CNN, the faster R-CNN, and the current state-of-the-art: the mask R-CNN. By using the more advanced model, the result should be lifted to the next level.

Summarizing the lesson learned from the related works, we notice that the radiography image has more minor features and patterns than the natural image. This implies that a thinner network structure may already be sufficient to complete the task. The observed knowledge inspired us to simplify the complexity of the VGG structure for our system.

## 3 Proposed Method

**FIGURE 1:**
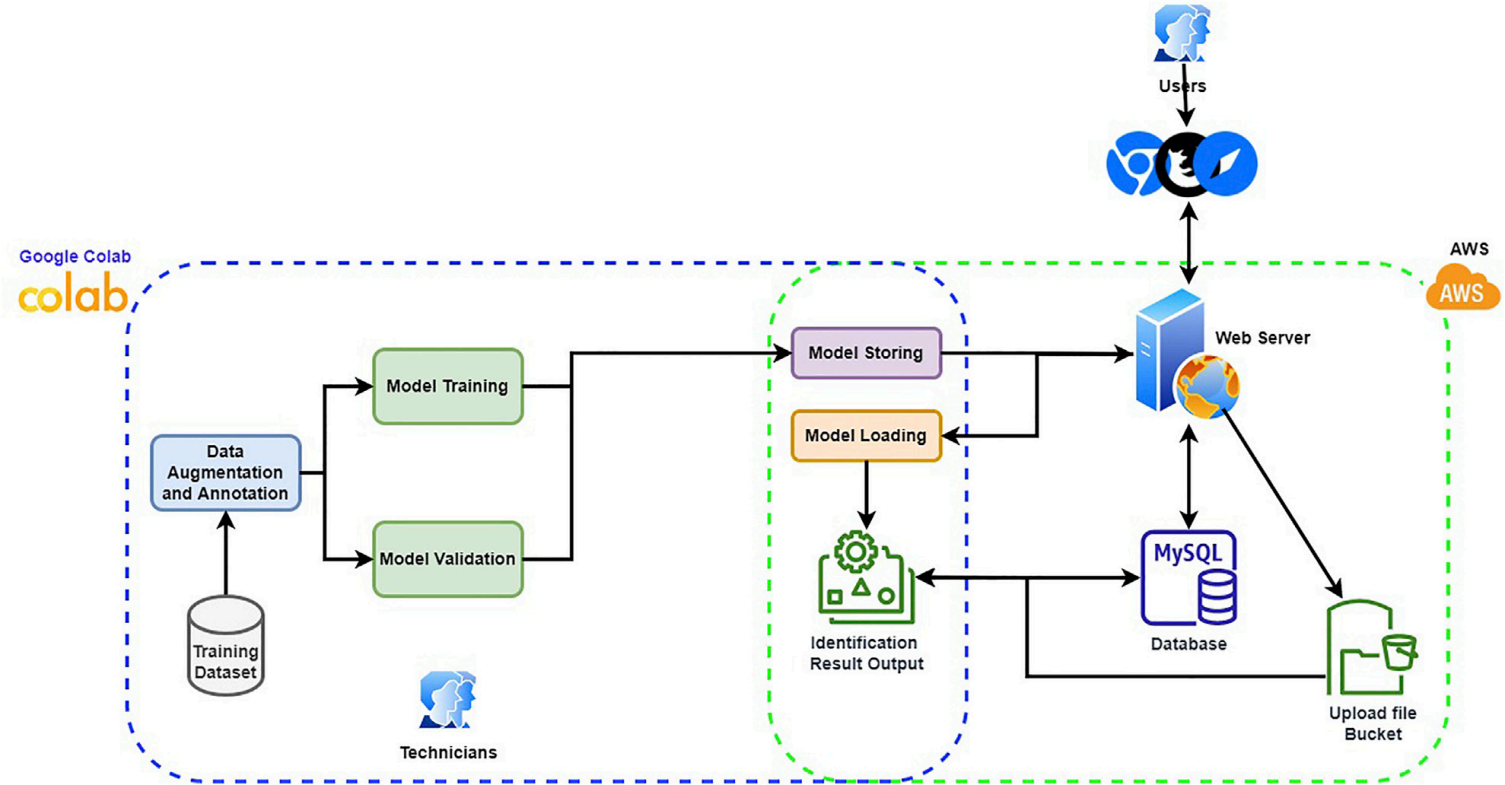
Proposed supporting system diagram.

Our goal in this work is to create a support system for the dentist to quickly identify the dental implant manufacturers from the x-ray image automatically. With the output from the support system, the dentist only needs to verify whether the result is correct rather than identifying the manufacturer with no reference information. Furthermore, considering the x-ray image is relatively monotone than the natural image, we expect a thinner network structure can accommodate the given task. Thus, we build a thinner VGG16 network called TVGG16 with a reduced number of neurons in the dense layers to reduce the computational cost. Figure 1 shows the diagram of the proposed dentist supporting system.

To make the system user friendly, the system is equipped with a graphical user interface (GUI), which is built by JavaScript with React plus the Flask API with Python. The model training is completed offline. The trained model is stored and loaded on the server. When the user inputs a dental x-ray image, the GUI offers cropping boxes for users to pinpoint the region of interest (RoI). The copped image is further resized to fit the model’s input and is uploaded from the client to the server for manufacturer identification. The identification results are collected from the enabled models, stacked up as the final result, and returned to the user with probabilities corresponding to different manufacturers. The back-end, including model building, training and test, are processed via Python with Google Colab as the IDE. Amazon Web Services (AWS) is used to host the server and the database. The database stores the information about the RoIs and the information will be used if a user latches a ticket for correcting the identification result. The server is used to store the trained models and to process the user-input test image. Users can hook up to the system via any web browser and connect to the web server to pass the submitted test image to the back-end process. The GUI guides the user through the process of choosing the test images and the RoIs. The identification result will then be returned and displayed in the browser. Since the built-in models are treated as on-demand modules to be loaded depending on whether the user chooses to include them in the identification process, the designed system can be easily scaled up by integrating new models in the back-end. More details of the components in the proposed system are given in the following subsections.

### 3.1 Graphical User Interface

**FIGURE 2:**
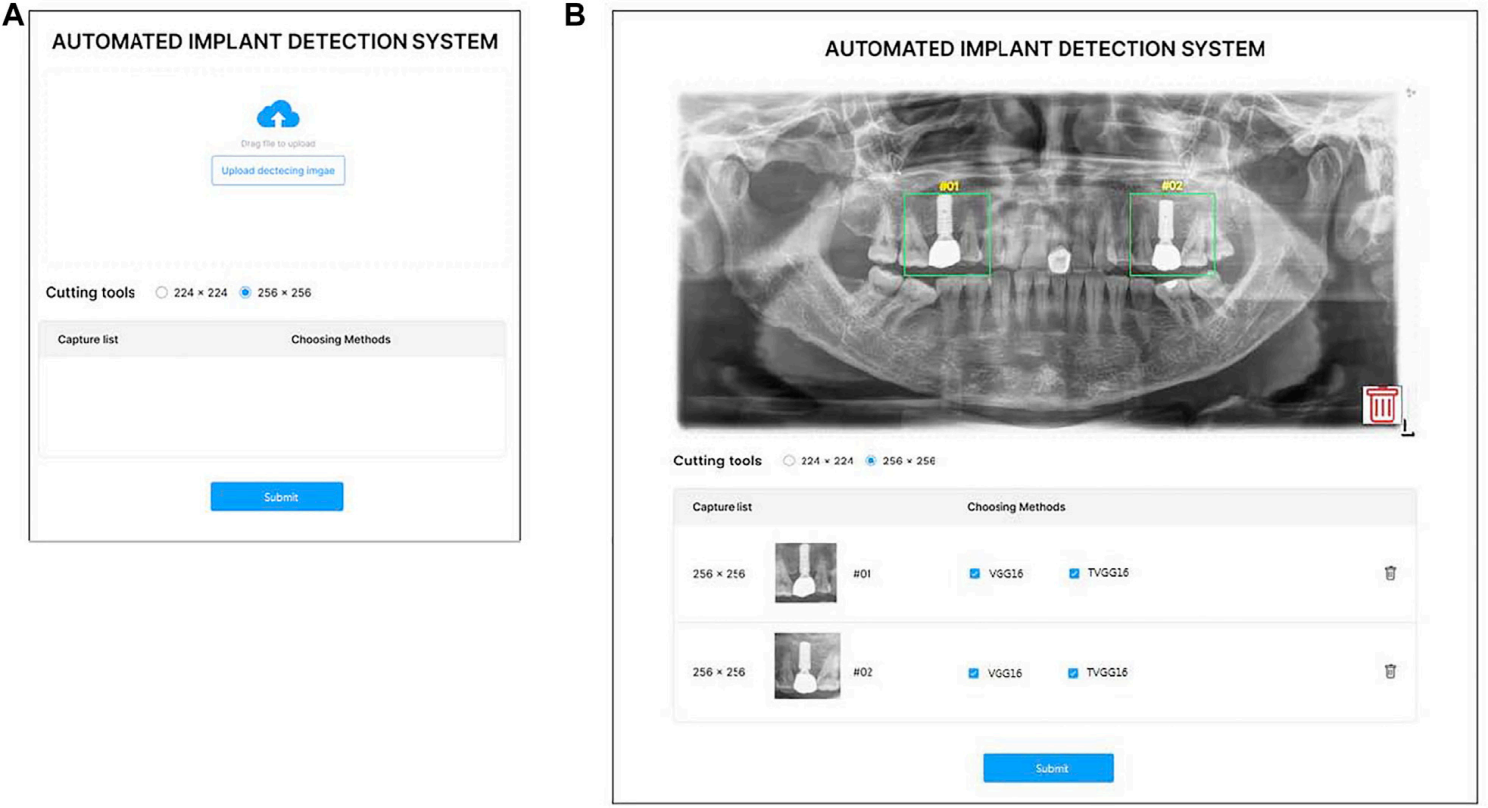
GUI of the proposed system **(A)** is the entry GUI **(B)** is the model selection and RoI specification GUI.

Considering the potential users of the proposed support system are not from the computer science background, a user-friendly interface is essential to lift the system’s usability. Hence, the system is designed with a GUI instead of a command line-based interface. Figure 2A demonstrates the GUI of the system for users to upload the x-ray image, and select the size of the bounding box. Figure 2B shows the GUI for users to indicate the RoI and select models. Two standard sizes, e.g. 224 × 224 and 256 × 256 of the RoI are built into the system to match the input image size of the pre-trained models. After uploading the x-ray image, users can drag on the interface to draw the user-defined RoIs. Once the RoIs are drawn, the cropping function is triggered automatically and resizes the RoIs to fit in the model input size. Users can then tick the boxes to select which models are employed in the identification process. Users can delete the RoIs or the uploaded image anytime before submitting them to the system by clicking the bin icon.

**FIGURE 3:**
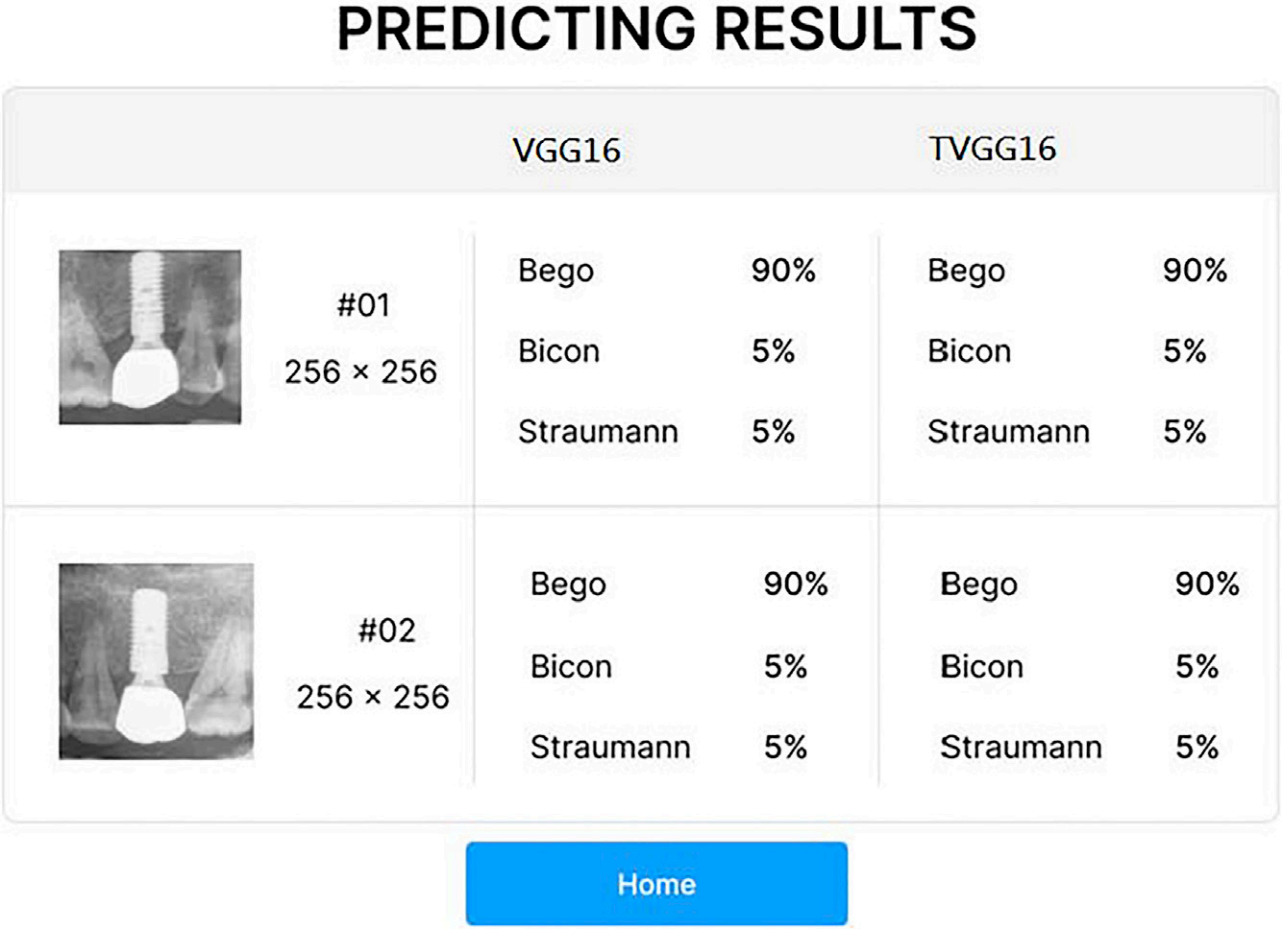
Result presentation GUI.

After the user clicks on the submission button, the RoIs are fed into the selected models on the server. After all models complete their prediction, the probabilities output from the selected models are returned for displaying on the system interface (see Figure 3). If the user clicks on the home button, the system returns to the entry page and stand-by to receive other dental x-ray images.

### 3.2 Thinner VGG (TVGG)

We choose VGG to be our base model for implementation because the existing study shows that VGG has more advantages in terms of energy consumption than other models (Canziani et al., 2017). As mentioned in Section 2, the monotone texture and patterns in the dental x-ray image caught our attention, and we assume that a simplified network structure is sufficient to cope with the implant manufacturer identification task. Nevertheless, adopting pre-trained models also has a strong point in reducing the training cost. If we remove layers in the model, we will need to start the training from scratch, which is much more resources and power-hungry than adopting the pre-trained model and performing fine-tuning. Aside from fine-tuning, we use the well-known dropout technique (Srivastava et al., 2014) as the tool to thinner the width of the dense layers. Dropout was originally proposed to prevent the network goes into overfitting. We adopt this technique but use its concept in removing neurons in the dense layers. Thus, this results in the dense layer in the modified VGG, e.g. the TVGG has much fewer neurons in the last few layers.

**FIGURE 4:**
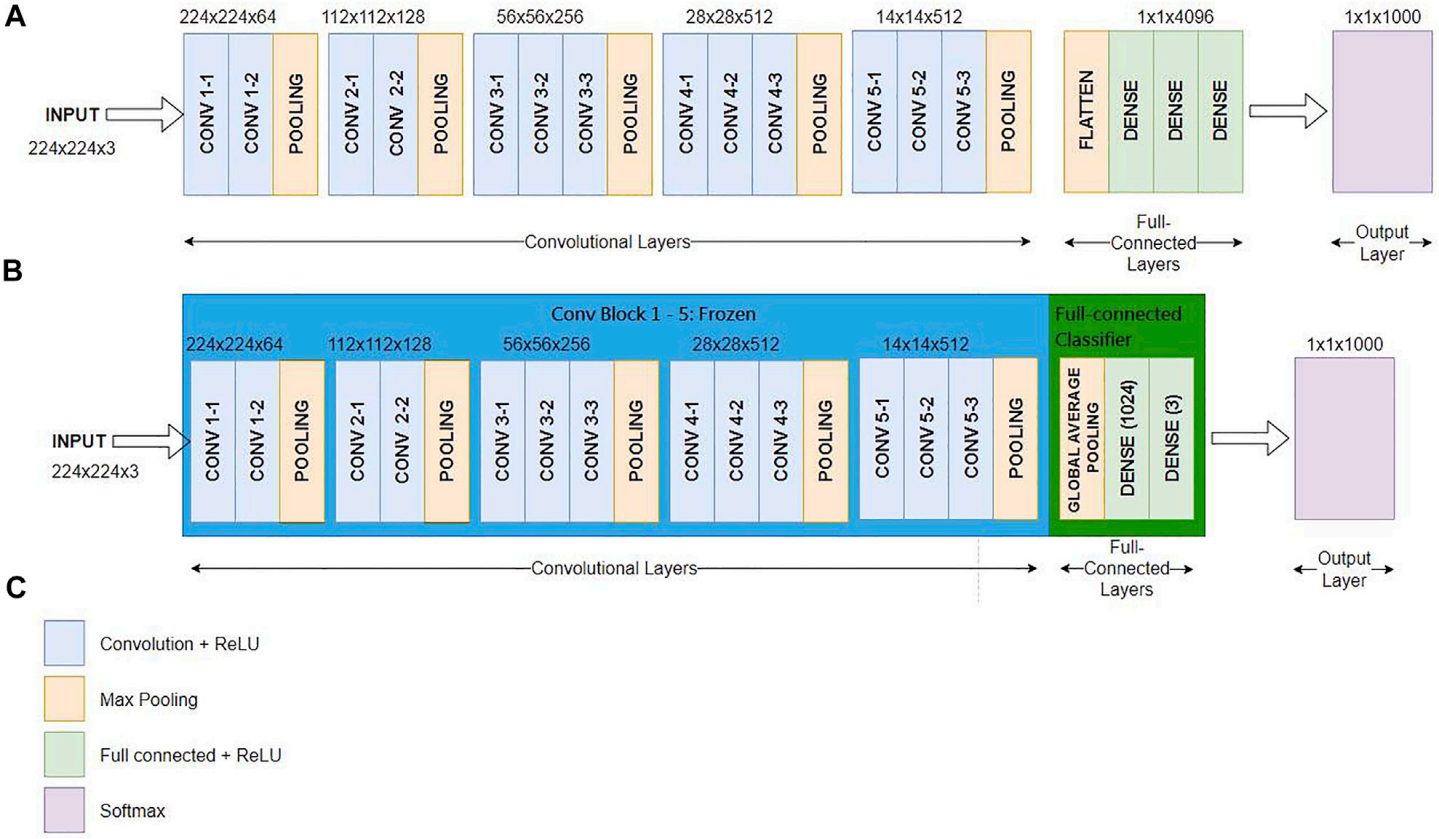
Network layout comparison **(A)** is the network layout of the conventional VGG16 **(B)** is the network layout of the TVGG **(C)** is the legend.

Figure 4A shows the conventional VGG16 layout (Simonyan and Zisserman, 2015), Figure 4B reveals our proposed TVGG layout, and Figure 4C presents the legend. Since the pre-trained VGG16 model is adopted as the base model, weights on the first-five convolution blocks are frozen in the training process. Only the weights on the full-connection layers are updated. Moreover, we replaced the original full connection layers from the flatten layer followed by three dense layers to the compact structure composed of the global average pooling and two dense layers. The dense layer size is also reduced by dropping out more than three-fourths of neurons.

## 4 Experiments and Result Discussions

The experiments are conducted in Python with Google Colab. The training environment contains two virtual CPUs, 24GB RAM, 150 GB storage space, and GPUs of K80, P100, and T4. Python libraries used in constructing the models include Tensorflow and Keras. Details of the experiment contents are described below.

### 4.1 Dataset

The dental x-ray images are collected at Fujian Medical University - Fujian Stomatological Hospital in P.R. China. It contains three classes of the dental implant manufacturer, namely, the Bego, the Bicon, and the Straumann, with 850, 892, and 527 x-ray images for the corresponding groups, respectively. This dataset is a hybrid of intraoral and extraoral x-ray images. Each of the classes contains both types of images. All collected x-ray images are containing implants from a single manufacturer. Mixture cases are not included in the collection.

### 4.2 Experiment Design

To ensure we have a balanced dataset across images from all manufacturers, data augmentation methods are used to expand the volume of the dataset to ensure all classes have the same quantity of data. Moreover, having data augmentation involved in the process helps avoid the overfitting issue. The methods used in the data augmentation process include a random rotation between $\left[-\frac{\pi }{9},\frac{\pi }{9}\right]$ degrees, object shifting in both vertical and horizontal directions within a 20% range, cropping and zooming both within 20% range, and horizontal flipping. With the help of the data augmentation, our model is capable of adopting the implant facing multiple directions. Thus the RoIs are not rotated to a specific angle in the test phase of our design. In the end, we have 550, 185, and 200 images for training, validation, and test, respectively, for each class.

The structure of the conventional VGG16 and our proposed TVGG is described in Section 3.2. We add another VGG16 model for comparison but replace the flatten layer with the global-average-pooling to have more models for comparison. The Adam optimization is used in all models in the training phase.

### 4.3 Evaluation Criteria

The common evaluation criteria in classification, including precision, recall, f1-score, and accuracy, are used to quantify the experimental results. Let *TP*, *FP*, *TN*, and *FN* represent the true positive, false positive, true negative, and false negative, respectively; these matrices can be calculated by Eqs 1–4.

$$\text{Precision}=\frac{TP}{TP+FP}$$ (1)

$$\text{Recall}=\frac{TP}{TP+FN}$$ (2)

$$F_{1}-\text{score}=\frac{TP}{TP+0.5\times \left(FP+FN\right)}$$ (3)

$$\text{Accuracy}=\frac{TP+TN}{TP+TN+FP+FN}$$ (4)

### 4.4 Experiment Results and Discussions

**FIGURE 5:**
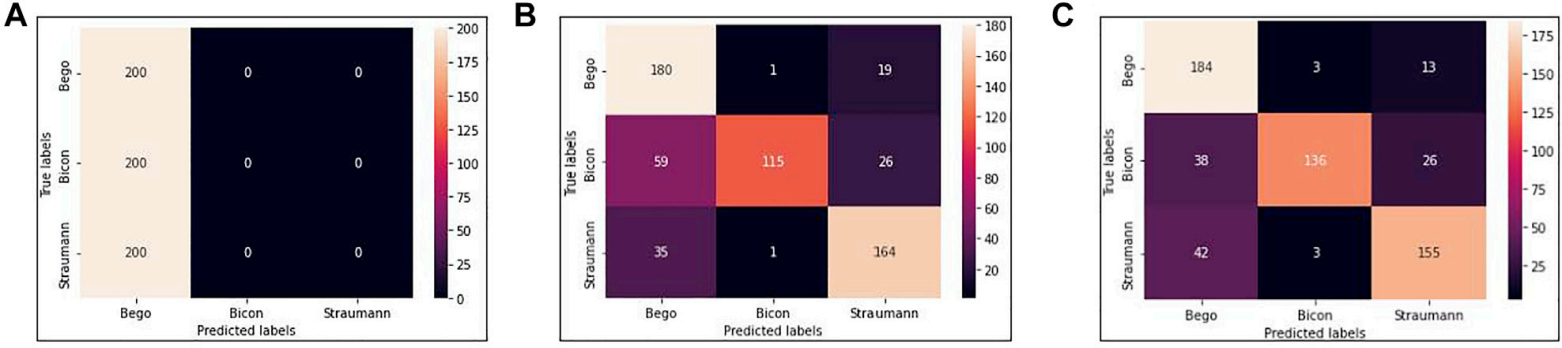
Confusion matrices **(A)** is obtained by VGG16 **(B)** is obtained by VGG16-GAP **(C)** is obtained by TVGG15.

We use VGG16, VGG16-GAP, and TVGG15 to indicate the conventional VGG16 model, the conventional VGG16 but replacing the flatten layer with global-average-pooling, and the thinner VGG, respectively. Figure 5 shows the confusion matrices from all models.

Our dataset size is relatively small compared to other studies. Figure 5A shows that using a small dataset to fine-tune the pre-trained VGG16 is not feasible because the training data volume is insufficient to support the tuning on a large scale of weights. Thus, we create the VGG16-GAP model to give the network a boost. From Figure 5B, the improvement is observable by the eye, and the correct classification results start to go across all classes. On the other hand, in the TVGG15 model, the correctly classified results (see Figure 5C) are getting higher than in the VGG16-GAP model.

Table 1 reveals the values from the evaluation matrices obtained by different implant manufacturers, namely, Bego, Bicon, and Straumann, respectively. The bold font marks the model performing the best in the column. It is observable that TVGG15 owns the most counts of achieving the best. The average performance across all classes is summarized in Table 2.

**TABLE 1:** Evaluation matrices of all class.

Model	Bego			Bicon			Straumann		
	Precision	Recall	F_1_-score	Precision	Recall	F_1_-score	Precision	Recall	F_1_-score
VGG16	0.33	**1.00**	0.50	0	0	0	0	0	0
VGG16-GAP	0.66	0.90	0.76	**0.98**	0.57	0.73	0.78	**0.82**	**0.80**
TVGG15	**0.70**	0.92	**0.79**	0.96	**0.68**	**0.80**	**0.80**	0.78	0.79

The bold values represents those presents the best across all methods.

**TABLE 2:** Model training information.

	Precision	Recall	F_1_-score
VGG16	0.11	0.33	0.17
VGG16-GAP	0.81	0.77	0.76
TVGG15	**0.82**	**0.79**	**0.79**

The bold values represents those presents the best across all methods.

**FIGURE 6:**
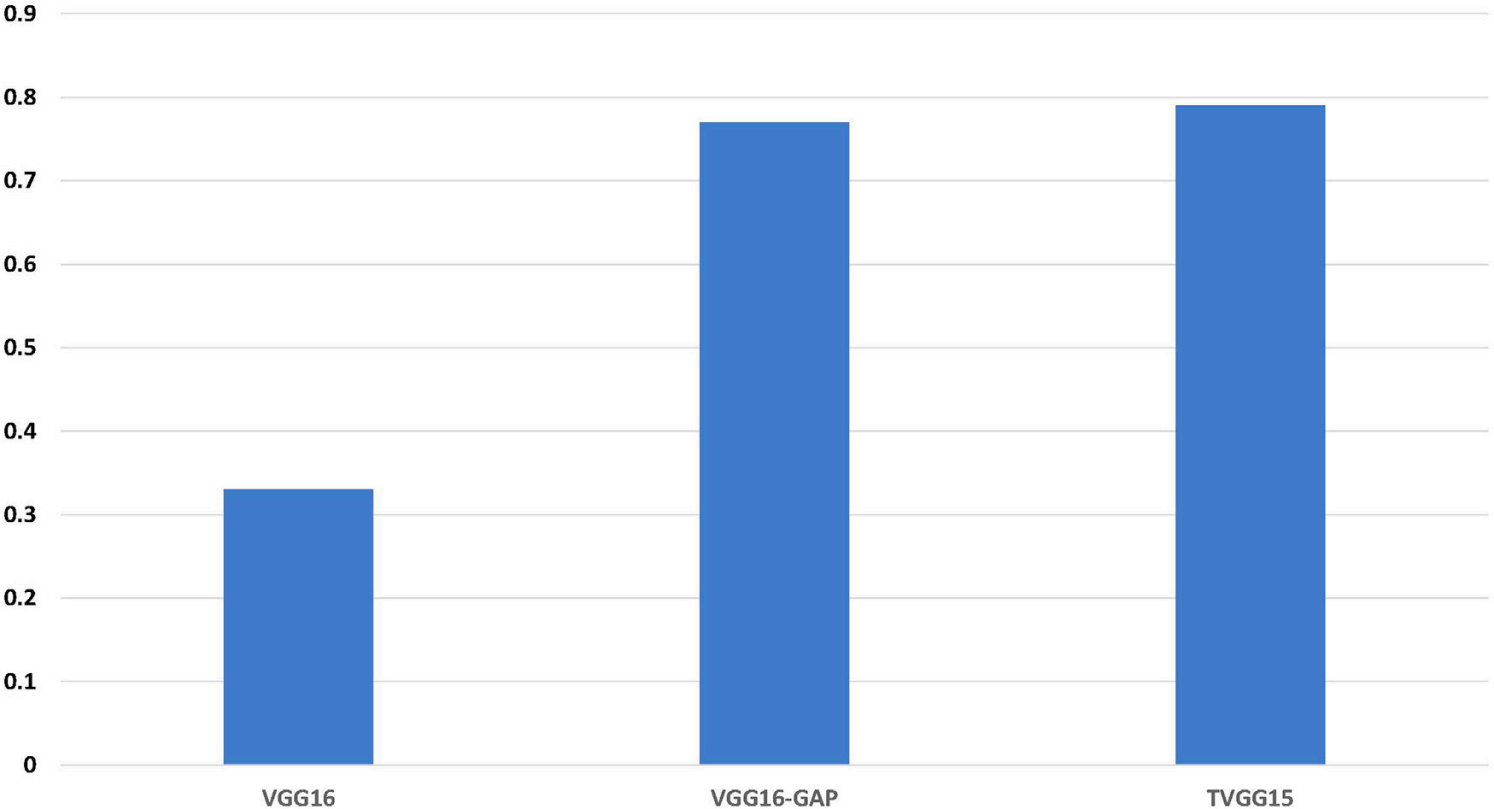
Identification accuracy across all models.

Figure 6 shows the accuracy calculated from the test results. TVGG15 presents the highest accuracy while VGG16-GAP achieves the second.

Table 3 shows the resource occupation, the training cost, and the training outcomes recorded by executing 200 epochs for each model. We can see that the TVGG15 model size is 2, 215 times less than VGG16 and 4.37 times less than VGG16-GAP. This compact characteristic gives TVGG15 an advantage in deploying the client-server internet environment. Moreover, by using the same resources for training the model, the training time of TVGG15 is reduced 4.3% than VGG16. The TVGG15 model also achieved the highest best training and validation accuracy.

**TABLE 3:** Model training information.

	VGG16	VGG16-GAP	TVGG15
Occupied Storage	1.38 GB	272.4 MB	**62.3** **MB**
Training Time (hours)	6.04	5.81	**5.78**
Best Training Accuracy	0.33	0.91	**0.92**
Best Validation Accuracy	0.33	0.88	**0.89**

The bold values represents those presents the best across all methods.

## 5 Conclusion

In this work, we create a dentist supporting system for automatic dental implant manufacturer identification from the dental x-ray images. The proposed system uses the TVGG15 model, which is an improved version from the VGG16 model pre-trained by ImageNet. The experiment results indicate that the pre-trained VGG16 is too large to be fine-tuned with the available quantity of data in our study. However, the proposed TVGG15 presents a satisfactory result in the case that the available training data is limited. Moreover, the proposed model occupies 2, 215 times less storage resource for preserving the model parameters given that the structure is not only more compact but also much thinner than the conventional VGG16 model. To gain the advantage of using a pre-trained model, the modification is only applied to the full connection layers in the CNN structure.

The dataset used in the experiment contains dental implants from a single manufacturer in each x-ray image, and thus no mixture case is included. However, our proposed system crops images into multiple RoIs. Even if there is an x-ray image containing a mixture of dental implants from numerous manufacturers, the proposed system should also present its performance as stable on the same level as in the current experiments.

In terms of building an application system, we see the potential to adopt the ensemble learning structure into the system back-end to increase the number of available candidate models in the future work. Heterogeneous models are more preferred because they can discover different characteristics of the input data. Moreover, we plan to remove the option for selecting the RoI cropping size in the GUI and only keep the one to a greater extent. Although different pre-trained models may require other input image sizes, the smaller RoI can be obtained by applying proper down-sampling techniques in the back-end and automatically satisfying this need. Furthermore, we plan to use some intelligent optimization methods to extend the modifiable parameters from the dense layers to the convolutional layers. This is a potential way to further imporve the performance of the models without retrain the whole models.

## Data Availability

The datasets presented in this article are not readily available because the patients' data is not publicly accessible. Further inquiries can be directed to the corresponding author JG, jianbin@fjmu.edu.cn.
